# Depolarization Controls TRAIL-Sensitization and Tumor-Selective Killing of Cancer Cells: Crosstalk with ROS

**DOI:** 10.3389/fonc.2014.00128

**Published:** 2014-05-30

**Authors:** Yoshihiro Suzuki-Karasaki, Miki Suzuki-Karasaki, Mayumi Uchida, Toyoko Ochiai

**Affiliations:** ^1^Division of Physiology, Department of Biomedical Sciences, Nihon University School of Medicine, Tokyo, Japan; ^2^Innovative Therapy Research Group, Nihon University Research Institute of Medical Science, Tokyo, Japan; ^3^Department of Dermatology, Nihon University Surugadai Hospital, Tokyo, Japan

**Keywords:** depolarization, ROS, TRAIL, tumor-selective killing, sensitization, apoptosis, oxidative stress, endoplasmic reticulum stress

## Abstract

Conventional genotoxic anti-cancer drugs target the proliferative advantage of tumor cells over normal cells. This kind of approach lacks the selectivity of treatment to cancer cells, because most of the targeted pathways are essential for the survival of normal cells. As a result, traditional cancer treatments are often limited by undesirable damage to normal cells (side-effects). Ideal anti-cancer drugs are expected to be highly effective against malignant tumor cells with minimal cytotoxicity toward normal cells. Such selective killing can be achieved by targeting pathways essential for the survival of cancer cells, but not normal cells. As cancer cells are characterized by their resistance to apoptosis, selective apoptosis induction is a promising approach for selective killing of cancer cells. Tumor necrosis factor-related apoptosis-inducing ligand (TRAIL) is a promising tumor-selective anti-cancer drug. However, the congenital and acquired resistance of some cancer cell types, including malignant melanoma cells, currently impedes effective TRAIL therapy, and an innovative approach that can override TRAIL resistance is urgently required. Apoptosis is characterized by cell shrinkage caused by disruption of the maintenance of the normal physiological concentrations of K^+^ and Na^+^ and intracellular ion homeostasis. The disrupted ion homeostasis leads to depolarization and apoptosis. Recent evidence suggests that depolarization is an early and prerequisite event during TRAIL-induced apoptosis. Moreover, diverse natural products and synthetic chemicals capable of depolarizing the cell membrane exhibit tumor-selective killing and TRAIL-sensitizing effects. Here, we discuss the role of depolarization in selective killing of cancer cells in connection with the emerging concept that oxidative stress is a critical mediator of mitochondrial and endoplasmic reticulum dysfunctions and serves as a tumor-selective target in cancer treatment.

## Introduction

Despite remarkable progress in cancer biology and treatment over the past 50 years, malignant neoplasms are still highly threatening diseases for humans, as they are frequently resistant to traditional chemotherapy, radiotherapy, and immunotherapy with a poor prognosis. Conventional genotoxic anti-cancer drugs target the proliferative advantage of tumor cells over normal cells. This kind of approach lacks the selectivity of treatment to cancer cells, because most of the targeted pathways are essential for the survival of normal cells. As a result, traditional cancer treatments are often limited by undesirable damage to normal cells (side-effects). Ideal anti-cancer drugs are expected to be highly effective against malignant tumor cells with minimal cytotoxicity toward normal cells. Such selective killing can be achieved by targeting pathways essential for the survival of cancer cells, but not normal cells. As cancer cells are characterized by their resistance to apoptosis, selective apoptosis induction is a promising approach for selective killing of cancer cells.

A growing list of natural products and synthetic chemicals exhibit tumor-selective killing effects (Tables 1 and 2). Under optimal conditions, they exhibit considerable cytotoxicity toward malignant cells, while sparing non-transformed cells. In addition, when applied at non-toxic concentrations, some of them can sensitize cancer cells to tumor necrosis factor (TNF)-related apoptosis-inducing ligand (TRAIL) cytotoxicity (Table 3). In fact, overlapping mechanisms appear to be involved in the tumor-selective killing and TRAIL-sensitizing effects, and the generation of intracellular reactive oxygen species (ROS) is the most common cellular event documented in the literature. An emerging view is that owing to their active metabolism and oncogenic stimulation, most cancer cells exhibit elevated ROS generation that makes them more vulnerable than normal cells to additional oxidative stress, and that such vulnerability can be exploited to selectively kill these cells. We will discuss this concept in more detail later. Although diverse natural and synthetic chemical compounds have been shown to possess selective killing effects, this review focuses on TRAIL, because TRAIL and its related substances are promising tumor-selective anti-cancer drugs that are currently undergoing clinical trials.

**Table 1:** **Natural products with tumor-selective cytotoxicity**.

Compounds	Cancer cells	Normal cells	Mode of cell death	ROS	Mechanisms of cell death	Reference
Wogonin	Leukemia	Lymphocytes	Apoptosis	H_2_O_2_	H_2_O_2_-PLC-Ca^2+^ overload-MMD-intrinsic death pathway	Baumann et al. (103)
					MMD	
					Involvement of VDCC in the Ca^2+^ response	
Wogonin	Hepatoma (HepG2)	Hepatic cells (LO2)	Apoptosis	${\text{O}}_{2}^{-}$, H_2_O_2_	ROS target Ca^2+^ release from ER (IP_3_-sensitive channels)-Ca^2+^ overload	Wei et al. (104)
					MMD, AIF release	
					Blockade by Bcl-2 (intrinsic death pathway)	
Wogonin	Glioma	Astrocytes	Apoptosis	H_2_O_2_ inhibition by apocynin	Intrinsic death pathway	Tsai et al. (105)
					ERS (GRP78, GRP94, calpain I, eIF2 phosphorylation)	
EGCG	Chondrosarcoma	Chondrocytes	Apoptosis	${\text{O}}_{2}^{-}$, H_2_O_2_	Upregulation of Bax, Bak	Yang et al. (106)
					Downregulation of Bcl-2, Bcl-xL	
					MMD, ASK1-p38/JNK pathway	
Capsaicin	Leukemia	T lymphocytes	Apoptosis	${\text{O}}_{2}^{-}$(outside of mitochondria)	Plasma membrane electron transport system	Macho et al. (107)
					MMD	
					Ca^2+^ mobilization (TRPV1)	
Capsaicin	Pancreatic cancer	HPDE-6	Apoptosis	H_2_O_2_	Complex I and III-mediated H_2_O_2_	Pramanik et al. (108)
					MMD	
					Cardiolipin oxidation	
					Intrinsic death pathway	
Pancratistatin	Neuroblastoma	Fibroblasts	Apoptosis	H_2_O_2_	Intrinsic death pathway	McLachlan et al. (109)
					MMD, ATP decrease	
Pancratistatin	Metastatic prostate cancer	Fibroblasts	Apoptosis	H_2_O_2_	Intrinsic death pathway	Griffin et al. (110)
			Autophagy		MMD	
Pancratistatin	Colorectal carcinoma	Fibroblasts	Apoptosis	Not determined	Bax, p53, caspase-independent death pathway	Griffin et al. (111)
					MMD	
					mtDNA-depleted cells are resistant	
Piperlongumine	Breast cancer, lung cancer, osteosarcoma	Fibroblasts, epithelial cells	Apoptosis	H_2_O_2_, NO	Transformation-associated ROS	Raj et al. (112)
					p53-Independent	
					Glutathione transferase-π/CBRl	
Diallyl sulfide (DAS)/diallyl disulfide (DADS)	Neuroblastoma	Neurons	Apoptosis	Not determined	Intrinsic death pathway	Karmakar et al. (113)
					Ca^2+^ mobilization	
					Increase in Bax/Bcl-2 ratio, Smac/Diablo release	
					Calpain activation, ICAD cleavage	
Resveratrol derivative	Prostate, colon cancer, hepatoma	Fibroblasts	Apoptosis	Not determined	Intrinsic death pathway	Gosslau et al. (114)
					Increase in Bax/Bcl-2 ratio, p53, Bax protein level	
					Perinuclear aggregation of mitochondria	
Bezielle	Breast cancer	Epithelial cells	Apoptosis	Mitochondrial ${\text{O}}_{2}^{-}$, H_2_O_2_	ROS-DNA damage-PARP hyperactivation-NAD/ATP depletion-glycolysis inhibition-energy collapse OXPHOS inhibition	Chen et al. (115)
bis-Dehydroxy-Curcumin	Colorectal carcinoma	Fibroblasts	Apoptosis	Not determined	Mitochondria-dependent apoptosis	Basile et al. (116)
			Autophagy		Caspase-7/8/9	
					ERS-induced autophagy	

**Table 2:** **Synthetic compounds with tumor-selective cytotoxicity**.

Compounds	Cancer cells	Normal cells	Mode of cell death	ROS	Mechanisms of cell death	Reference
Glitazones	Glioma	Astrocytes	Apoptosis	${\text{O}}_{2}^{-}$, NO, ONOO^−^	MMD	Pérez-Ortiz et al. (117)
					Reduction in mitochondrial pH	
					ETC complex I	
Salinomycin	Prostate, breast cancer	Fibroblasts	Autophagy (cell protective)	Not determined	Increased mitochondrial mass	Jangamreddy et al. (118)
					MMD, MHP (subpopulation)	
			Apoptosis		Caspase-3, -8, -9	
			Necrosis		ATP decrease	
					Mitochondrial fragmentation	
					Mitophagy	
					Mitoptosis	
Rosiglitazone ciglitazone (PPARγ ligands)	Glioma	Astrocytes	Apoptosis	H_2_O_2_	PPARγ-independent	Pérez-Ortiz et al. (119)
					MMD	
					Inhibition by ebselen, NAC	
Rotenone/TT FA	Glioma	Astrocytes	Apoptosis	Mitochondrial ${\text{O}}_{2}^{-}$	Complex I and II-mediated ROS-autophagy	Chen et al. (120)
			Autophagy	
2-Methoxy-estradiol	Glioma	Astrocytes	Autophagy	${\text{O}}_{2}^{-}$	Apoptosis-independent	Chen et al. (121)
					ROS-mediated autophagy	
GYY4137	Hepatoma, leukemia, colorectal carcinoma, etc.	Fibroblasts	Apoptosis	(H_2_S)	Intrinsic death pathway	Lee et al. (122)

**Table 3:** **Reactive oxygen species-generating TRAIL sensitizers**.

Compounds	Action	Target cells	Mode of cell death	ROS	Mechanisms of sensitization	Reference
CCCP	OXPHOS uncoupler	Colon carcinoma	Apoptosis	${\text{O}}_{2}^{-}$	Caspase-dependent	Izaradjene et al. (67)
					MMD	
					ROS-dependent Bax and caspase-8 activation	
					Inhibition by Bcl-2, XIAP	
					Caspase-9-independent	
LY303511	Inactive LY294402 analog	Neuroblastoma	Apoptosis	H_2_O_2_	H_2_O_2_-induced JNK/ERK-mediated upregulation of DR4 and DR5	Shenoy et al. (49)
Selenium	Chemopreventive agent	Prostate cancer	Apoptosis	${\text{O}}_{2}^{-}$	${\text{O}}_{2}^{-}$ mediated p53 Ser^15^	Hu et al. (123)
					Phosphorylation	
					Bax increase and translocation	
Wogonin	Anti-cancer agent	Prostate cancer	Apoptosis	H_2_O_2_	${\text{O}}_{2}^{-}$ mediated upregulation of p53 and Puma	Lee et al. (50)
KCl/U37883A/glibenclamide	PMD	Melanoma, leukemia, lung cancer	Apoptosis	Mitochondrial ${\text{O}}_{2}^{-}$	PMD	Suzuki et al. (19), Suzuki-Karasaki et al. (21)
	ATP-sensitive K^+^ channel inhibitors				MMD	
					Upregulation of DR4 and DR5 ERS (XBP-1, caspase-12)	
DATS	Anti-cancer agent	Melanoma	Apoptosis	H_2_O_2_, ${\text{O}}_{2}^{-}$	Upregulation of DR4 and DR5	Murai et al. (22)
					PMD, MMD	
					ERS (XBP-1, caspase-12)	
H_2_O_2_	Membrane-permeable ROS	Melanoma	Apoptosis	Mitochondrial ${\text{O}}_{2}^{-}$	Upregulation of DR4 and DR5	Tochigi et al. (23)
					PMD, MMD	
					ERS (XBP-1, caspase-12)	
Rotenone, antimycin A, FCCP	OXPHOS inhibitor	Melanoma, lung cancer	Apoptosis	Mitochondrial ${\text{O}}_{2}^{-}$	PMD, MMD	Inoue and Suzuki-Karasaki (20)

## TRAIL and Cancer Cell Death

Tumor necrosis factor-related apoptosis-inducing ligand is a member of the TNF cytokine family. It binds to several distinct receptors, death receptor (DR) 4/TRAIL receptor 1 (TRAIL-R1), DR5/TRAIL-R2, TRAIL-R3, and TRAIL-R4 (1). Both DR4 and DR5 contain an intracellular death domain that is essential for the induction of apoptosis following receptor ligation. In contrast, despite their binding to TRAIL, neither TRAIL-R3 nor TRAIL-R4 mediates apoptosis owing to a complete or partial lack of the intracellular death domain. These receptors are regarded as decoy receptors (DcRs) (1, 2). TRAIL activates the extrinsic apoptotic pathway. Binding of TRAIL to DR4/DR5 induces their oligomerization and conformational changes in their death domains, resulting in the formation of a death-inducing signaling complex and subsequent activation of the initial caspase, caspase-8. In turn, activated caspase-8 activates the effector caspases, caspase-3/6/7 that execute the apoptotic process (3, 4). Activation of caspase-8 is also linked to the intrinsic (mitochondrial) apoptotic pathway. Activated caspase-8 can cleave and activate the pro-apoptotic Bcl-2-family molecule Bid. In turn, truncated Bid activates other Bcl-2-family molecules, Bax and Bak, resulting in their oligomerization and the formation of megachannels in the outer mitochondrial membrane (OMM). The release of cytochrome *c* through these Bax/Bak megachannels into the cytosol induces assembly of the apoptosome, representing the activation-platform for another initial caspase, caspase-9. Activated caspase-9 also promotes the activation of caspase-3/6/7, thereby providing a positive loop for caspase activation (3). Unlike TNF-α, TRAIL has been shown to induce apoptosis in cancer cells with minimal cytotoxicity toward non-transformed cells (4), although under certain circumstances, it enhances the cytotoxicity of several drugs to hepatocytes/liver and mast cells (5–9). Thus, TRAIL is a promising agent in cancer treatment with high selectivity. However, different cancer cell types such as malignant melanoma, glioma, and non-small cell lung cancer (NSCLC) cells are resistant to TRAIL treatment despite expressing DRs on their cell surface. Moreover, TRAIL-responsive tumors acquire a resistant phenotype that renders TRAIL therapy ineffective (10). Therefore, overcoming TRAIL resistance is necessary for effective TRAIL therapy, and small molecules that can potentiate TRAIL effectiveness are urgently required. Recently, much progress has been made in therapeutic intervention with TRAIL-related substances. Recombinant human TRAIL (rhTRAIL) or agonist monoclonal antibodies (mAbs) against DR4/DR5, collectively referred as to pro-apoptotic receptor agonists (PARAs), have been subjected to clinical trials for a variety of cancer cell types, including malignant melanoma and NSCLC cells. In addition, diverse chemical substances including ABT-737 and SM-164 are expected to potentiate the intrinsic death pathway by antagonizing natural inhibitors such as FLICE inhibitory protein, Bcl-2, Mcl-1, and survivin (11). Some recent clinical trials have demonstrated the safety and efficiency of combined treatments with PARAs and conventional genotoxic drugs, while the results of other clinical trials were disappointing and showed only modest effectiveness. PARAs such as dulanermin (rhTRAIL), mapatumumab (anti-DR4 mAb), conatumumab, CS-1008, or PRO95780 (anti-DR5 mAb), in combination with paclitaxel, carboplatin, or bevacizumab [anti-vascular endothelial growth factor (VEGF) mAb] were tested for their effects toward NSCLC patients in a randomized phase II trial, but showed only modest effects (11). Thus, induction of apoptosis by the intrinsic pathway does not appear to be a suitable target in the treatment of TRAIL-resistant cancer cells.

## Dual Function of Depolarization in the Regulation of Apoptosis

Apoptosis is a fundamental physiological process characterized by the loss of cell volume (cell shrinkage), chromatin condensation, and internucleosomal DNA fragmentation. Cell shrinkage is a hallmark of apoptosis, which is caused by disruption of the maintenance of normal physiological concentrations of K^+^ and Na^+^ and intracellular ion homeostasis (12, 13). Loss of these monovalent ions has been reported to facilitate the loss of cell volume (apoptotic volume decrease) and caspase-3 activation (13). Cell shrinkage requires ion transport activity across the cell membrane, including Cl^−^ and K^+^ channels. Impairment of ion channels or transporters responsible for Na^+^, K^+^, Cl^−^, and Ca^2+^ can disrupt intracellular ion homeostasis and lead to cell membrane depolarization and apoptosis. In fact, depolarization has been shown to be an early event in the apoptosis induced by diverse agents, including Fas, rotenone, and arsenic trioxide (14–16), and is considered to play an important pro-apoptotic role. In contrast, depolarization has also been shown to exhibit anti-apoptotic effects. Various membrane-depolarizing agents, including ouabain, tetraethylammonium (TEA), and veratridine, protect Purkinje cells against apoptosis (17). In addition, K^+^ loading and several K^+^ channel inhibitors protect various human tumor cells against staurosporine-induced apoptosis. Thus, depolarization can act in both pro-apoptotic and anti-apoptotic manners depending on the cell types and apoptotic stimuli involved. There is no general model that can depict the dual functions of depolarization.

## Na^+^–K^+^-ATPase is a Key Player Connecting Death Ligands, Depolarization, and Apoptosis

The Na^+^ pump Na^+^–K^+^-ATPase mediates the export of three Na^+^ ions the import of two K^+^ ions, thereby maintaining high K^+^ and low Na^+^ intracellular concentrations. Diverse apoptotic stimuli including anti-Fas, A23187, and thapsigargin have been shown to induce depolarization without repolarization in Jurkat cells (14). This response is rapid (1–2 h after stimulation) and observed in a time-dependent manner. Unlike the depolarization observed in electrically excited cells, this apoptosis-associated depolarization is rather sustained (observed for throughout at least 8 h), as the depolarization lacks repolarization. These effects can be accounted for inactivation of Na^+^–K^+^-ATPase protein and its activity. Persistent depolarization caused by impairment of Na^+^–K^+^-ATPase is also associated with other apoptotic stimuli such as the mitochondrial toxins rotenone and squamocin (15). The persistent depolarization is observed not only in human leukemia cells such as Jurkat cells and U937 cells but also in human primary T cells, although primary cells are more resistant than leukemia cells to the effect. The persistent depolarization is pro-apoptotic, because some cardiac glycoside inhibitors of Na^+^–K^+^-ATPase such as ouabain and oleandrin, can sensitize human leukemia cells and NSCLC cells to apoptosis induced by anti-Fas, TRAIL, and mitochondrial toxins (14, 15, 18). These observations suggest that Na^+^–K^+^-ATPase is a key player connecting death ligands, depolarization, and apoptosis.

## Role of Depolarization in TRAIL-Induced Apoptosis of Malignant Tumor Cells

Our recent work has shown that persistent depolarization also occurs in the early stage of TRAIL-induced apoptosis in malignant tumor cells. TRAIL dose- and time-dependently induces robust depolarization in human malignant tumor cells, including melanoma, leukemia, and lung cancer cells after a time lag of 2-4 h (19, 20). In all tumor cell lines tested, the magnitude of depolarization is correlated with their sensitivity to TRAIL. Importantly, the depolarization appears to be a prerequisite event for TRAIL-induced apoptosis, because TRAIL induces minimal depolarization in normal melanocytes, to which it shows minimal cytotoxicity despite their substantial cell surface expression of DR4 and DR5 (19). The pro-apoptotic role of depolarization is supported by the finding that high K^+^ loading sensitizes melanoma cells, but not melanocytes, to TRAIL. As ATP-sensitive K^+^ (K_ATP_) channels mediate the efflux of intracellular K^+^ into the cytosol, thereby promoting plasma membrane hyperpolarization, inhibition of these channels is expected to induce depolarization and show similar effects. In fact, selective inhibitors of K_ATP_ channels such as glibenclamide and U37883A show similar TRAIL-sensitizing effects, while TEA, which mainly inhibits voltage-dependent K^+^ (K_v_) and calcium-dependent K^+^ (K_Ca_) channels, is ineffective, suggesting a specific role of K_ATP_ channels in the potentiation. In support of this view, the K_v_ channel-specific inhibitor α-dendrotoxin, K_Ca_ channel-specific inhibitor charybdotoxin, and mitochondrial K_ATP_ channel inhibitor 5-hydroxydecanoate have no such effects in melanoma and leukemia cells (19–21). As discussed below, the depolarization-mediated potentiation of apoptosis is associated with activation of the mitochondrial apoptotic pathway and intracellular ROS generation. Consistent with this view, glibenclamide has been shown to exert antitumor activity in human gastric cancer cells through the intrinsic pathway and ROS generation. Since Na^+^–K^+^-ATPase seems to play a key role in controlling death ligand-induced depolarization and apoptosis, the enzyme may also be involved in the depolarization induced by TRAIL. However, it is noted that among these death ligands, only TRAIL exerts tumor-selective cytotoxicity. Nonetheless, cardiac glycoside Na^+^–K^+^-ATPase inhibitors such as oleandrin (18) and K_ATP_ channel inhibitors share some biological effects including upregulation of DR4 and DR5. Thus, further studies are necessary to define the role of Na^+^–K^+^-ATPase in TRAIL-induced apoptosis in human cancer cells.

## Role of the Mitochondrial Death Pathway in the Effects of Depolarization

In addition to high K^+^ and K_ATP_ channel inhibitors, different natural and synthetic chemicals have recently been identified as powerful potentiators of TRAIL-induced apoptosis in malignant melanoma cells regardless of their diverse chemical structures and biological targets. These include diallyltrisulfide (DATS), a major garlic organosulfur compound (22), the cell-permeable oxidant H_2_O_2_ (23), and mitochondrial metabolic inhibitors such as rotenone, antimycin A, and FCCP (20). Moreover, when employed at higher concentrations and longer exposures, all of these chemicals kill malignant cells while sparing normal cells. Strikingly, all of these agents also can commonly induce robust depolarization prior to apoptosis, supporting a universal role for depolarization in the potentiation of TRAIL-induced apoptosis and tumor-selective killing. In contrast, agents that do not affect cell survival such as TEA, have no effect on DR5 expression, suggesting that upregulation of DR5 expression is a common target of these membrane-depolarizing agents for potentiating apoptosis. This view is also in agreement with previous studies demonstrating that amplification of TRAIL-induced apoptosis by diverse agents, including thapsigargin, tunicamycin, and 2-deoxy-d-glucose, is associated with upregulation of DR5 expression (24–26). The intrinsic mitochondrial pathway plays a crucial role in amplifying TRAIL-induced apoptosis, and collapse of the mitochondrial membrane potential (ΔΨ_m_) is considered to be a hallmark of this pathway, although it remains a matter of debate whether this event is a cause or a result of permeabilization of the OMM (27, 28). Permeabilization of the OMM by pro-apoptotic Bcl-2 family proteins promotes the release of a number of apoptogenic factors, such as cytochrome *c*, endonuclease G, second mitochondrial activator of caspases (SMAC), Omi/HtrA2, and apoptosis-inducing factor (AIF), from the inner mitochondrial membrane (IMM) space into the cytosol, and these apoptogenic proteins promote activation of the caspase cascade, thereby leading to apoptosis. Cytochrome *c* interacts with the apoptotic peptidase activating factor 1 (Apaf-1), leading to the formation of the multimeric apoptosome in the presence of ATP/dATP (29). The apoptosome acts as a platform for activation of the initiator caspase, caspase-9, which subsequently cleaves and activates the effector caspases, caspases-3 and -7. A cytochrome *c*-independent apoptosis pathway has also been defined, and this pathway requires proteins such as endonuclease G and AIF to carry out apoptosis. Although the molecular mechanisms underlying the OMM permeabilization are poorly understood, it is widely accepted that the mitochondrial permeability transition (MPT), which was originally defined as a sudden increase in IMM permeability to solutes with molecular masses of ~1,500 Da, is involved. It is now believed that opening of putative megachannels referred to as the mitochondrial permeability transition pores (mPTPs) plays pivotal roles (30, 31). mPTPs are high-conductance non-specific pores in the IMM composed of proteins that link the IMM and OMM. Several mitochondrial proteins localized in the IMM and OMM, such as voltage-dependent anion channels (VDACs), adenine nucleotide translocase (ANT), hexokinase, peripheral benzodiazepine receptors, and cyclophilin-D are thought to constitute the mPTP. Under physiological conditions, the proteins in the OMM and IMM that constitute mPTPs are thought to be in close proximity to one another and in a closed or low-conductance conformation, although mPTPs have not been isolated and the components of these complexes remain controversial (32–34). When an mPTP changes to an open conformation, water and solutes with molecular masses of up to 1,500 Da enter the mitochondrial matrix, resulting in osmotic swelling of the mitochondrion. It is thought that when multiple mPTPs open concurrently and extensive mitochondrial swelling takes place, physical disorganization of the OMM occurs and mitochondrial apoptogenic proteins are released, thereby triggering apoptosis (27). Hence, much attention has been paid to the potential role of mPTPs as a target for anti-cancer and chemopreventive agents (27, 28). TRAIL induces both ΔΨ_m_ collapse and caspase-3/7 activation in melanoma and leukemia cells and the sensitization of TRAIL-induced apoptosis by membrane-depolarizing agents is associated with their enhancement, indicating the involvement of the intrinsic pathway. Although glibenclamide and U37883A are potent K_ATP_ channel inhibitors, glibenclamide targets sulfonylurea receptors (SURs) while U37883A is non-SUR drug. It is noted that glibenclamide and U37883A have different effects on TRAIL-induced cell death depending on TRAIL sensitivity. In TRAIL-resistant A375 melanoma cells, U37883A can potentiate TRAIL-induced cell death as rapidly as within 24 h, while glibenclamide is ineffective, despite considerable potentiation of TRAIL-induced ΔΨ_m_ collapse and caspase-3/7 activation. This cell death is mainly caused by apoptosis. However, after 72 h of treatment, glibenclamide alone can induce considerable apoptotic and necrotic cell death and potentiates TRAIL effectiveness. However, both U37883A and glibenclamide can enhance apoptosis in TRAIL-sensitive Jurkat leukemia cells during the initial 24 h (20). Thus, these observations suggest that: (i) TRAIL and the two K_ATP_ channel inhibitors can induce different modes of cell death depending on the basal cellular sensitivity to TRAIL and experimental conditions such as exposure time, which is supported by the fact that substantial necrotic cell death is associated with the late cell death, but not the early cell death; (ii) SURs may play differential roles in the two modes of cell death in TRAIL-resistant cells; and (iii) activation of mitochondrial death pathway is insufficient for complete overriding of TRAIL resistance in malignant cells. This view coincides with the findings in clinical trials that the combined use of PARAs and other conventional chemotherapeutic drugs exhibit only modest effects toward TRAIL-resistant cancers, although they can induce substantial activation of the intrinsic death pathway (11). Consequently, the potentiation of TRAIL-induced apoptosis in TRAIL-resistant cancer cells by membrane-depolarizing agents may involve another cell death pathway.

## Role of the Endoplasmic Reticulum Death Pathway in the Effects of Depolarization

The emerging concept is that, besides mitochondria, the endoplasmic reticulum (ER) is another key player in the regulation of apoptosis induced by a variety of death stimuli, including TRAIL. Disparate perturbations in their normal ER functions, such as accumulation of unfolded or misfolded proteins, ER lipid imbalances or changes in the redox balance, or Ca^2+^ conditions in the ER lumen, trigger ER stress (ERS) (35–38). The cells then activate ERS responses, called the unfolded protein response (UPR), to alleviate the stress, but an excessive and prolonged UPR leads to apoptosis (36–38). The UPR involves transcription-dependent upregulation of ER-resident chaperones, and the ER chaperone glucose-related protein 78 (GRP78; also known as Bip) is thought to be a primary sensor in this response. Upon ERS, GRP78 dissociates from ER transmembrane proteins, such as inositol requiring enzyme 1 (IRE1) and activating transcription factor 6 (ATF6), to bind to unfolded or misfolded proteins, resulting in aggregation of the transmembrane proteins and their activation. Activated IRE 1 splices the mRNA for X-box-binding protein-1 (XBP-1) to allow translation of the mature spliced form of XBP-1 protein, which acts as a transcription factor and mediates the transcriptional upregulation of numerous genes involved in ER function as well as TRAIL-R2 (25). In support of the role of ERS in TRAIL-induced apoptosis, TRAIL induces the splicing of XBP-1, and membrane-depolarizing agents including high K^+^, U37883A (19, 21), DATS (22), H_2_O_2_ (23), and mitochondrial inhibitors such as antimycin A and FCCP (20) all commonly potentiate this effect, although none of these agents except H_2_O_2_ alone can induce the splicing. Only glibenclamide cannot potentiate TRAIL-induced XBP-1 activation, although it upregulates GRP78 expression in accordance with its minimal effect on TRAIL-induced cell death during the initial 24 h (19). These observations are interesting because GRP78 acts as an anti-apoptotic factor in the cells, since downregulation of GRP78 expression by small interfering RNA (siRNA) administration potentiates the apoptosis induced by diverse drugs including cisplatin, adriamycin (25), fenretinide [*N*-(4-hydroxyphenyl) retinamide], a synthetic derivative of retinoic acid, and bortezomib, a 26S proteasome inhibitor (39). Taken together, it is strongly suggested that pro-apoptotic ERS responses including GRP78 downregulation, XBP-1 upregulation and processing are activated by depolarization and play an important role in TRAIL-sensitization and tumor-selective killing.

## Dual Role of ROS in Apoptosis

Reactive oxygen species, such as superoxide anions (${\text{O}}_{2}^{•-}$), H_2_O_2_, and hydroxyl radicals (•OH), are the products of normal metabolism in virtually all aerobic organisms. Low physiological levels of ROS function as second messengers in intracellular signaling and are required for normal cell function, while excessive ROS cause damage to multiple macromolecules, impair cell function, and promote apoptotic or necrotic cell death including those induced by DR ligands (40, 41). ROS levels are controlled by the antioxidant defense system, including the antioxidant enzymes manganese- or copper–zinc-containing superoxide dismutase (SOD), which catalyzes the dismutation of ${\text{O}}_{2}^{•-}$ into H_2_O_2_, and catalase and glutathione peroxidase, which degrade H_2_O_2_. Thus, the balance between the machinery for ROS generation (prooxidant system) and the machinery for ROS scavenging (antioxidant system) is a critical determinant of whether the cell fate is proliferation or death. Indeed, generation of ROS is also associated with the apoptosis induced by DR ligands such as Fas (42–45) and TNF-α (46, 47). TRAIL has been shown to induce the generation of intracellular ROS, including H_2_O_2_, which may be critical in regulating the responses of cancer cells to TRAIL (48). A variety of natural and synthetic compounds capable of increasing the intracellular H_2_O_2_ level, including LY35001 (49), wogonin (50), and diallyl polysulfides (51, 52) potentiate TRAIL cytotoxicity toward different human malignant cells (Table 3). Moreover, direct application of H_2_O_2_ alone induces apoptosis or potentiates death ligand-induced apoptosis in different cell types (23, 53). In addition, various antioxidants such as *N*-acetyl-l-cysteine (NAC) and manganese SOD, and catalase block TNF-α- and Fas-induced apoptosis (54, 55). These observations suggest that ROS are important mediators of death ligand-induced apoptosis. Both NADPH oxidase (42, 45, 47) and mitochondria (43, 46) have been implicated as the cellular sources of ROS generated by TNF-α- or Fas-mediated signaling in various cell types. Conversely, ROS or prooxidative conditions are protective under certain circumstances (56). Thus, ROS seem to exhibit both pro-apoptotic and anti-apoptotic functions. Hence, the role of H_2_O_2_ in DR-mediated cell death remains controversial. To date there is no general model that can explain the dual role of ROS in apoptosis. However, accumulating evidence suggests that ROS control another form of cell death, autophagy (57, 58), which protects cancer cells against apoptosis. This emerging concept is interesting because it may explain that ROS exert their anti-apoptotic function through the control of cancer cell autophagy. However, this issue is not the focus of the present review, as it has been comprehensively reviewed by others (59–61).

## Role of Mitochondrial ${\text{O}}_{2}^{•-}$ in Cancer Cell Apoptosis

Another possible explanation for the dual role of ROS in TRAIL-induced apoptosis is that different oxidant species mediate reciprocal effects. Indeed, the generation of various ROS and reactive nitrogen species including H_2_O_2_, ${\text{O}}_{2}^{•-}$, nitric oxide (NO), and peroxynitrite (ONOO^−^) is associated with selective killing (Tables 1 and 2) and TRAIL-sensitization (Table 3) in cancer cells. To define the oxidant species mediating TRAIL-induced apoptosis in cancer cells, we analyzed ROS generation using 2′,7′-dichlorodihydrofluorescein diacetate (DCFH-DA) and dihydroethidine (DHE) after TRAIL treatment. DCFH-DA is rapidly taken up by cells and hydrolyzed into DCFH by cellular esterase activity. DCFH is then converted into the fluorescent compound DCF by oxidation mediated by H_2_O_2_, ONOO^−^, and •OH, but not ${\text{O}}_{2}^{•-}$. Conversely, DHE undergoes two-electron oxidation to form DNA-binding ethidium bromide in a reaction that is relatively specific for ${\text{O}}_{2}^{•-}$. Consequently, DCFH-DA and DHE have been widely used to detect intracellular H_2_O_2_ and ${\text{O}}_{2}^{•-}$, respectively in various cell types (62–64). TRAIL treatment results in the generation of intracellular H_2_O_2_ and ${\text{O}}_{2}^{•-}$ in Jurkat leukemia and A375 melanoma cells (20). The time courses of the generation of these two oxidants are different in that the H_2_O_2_ generation is rapid (detected within 30 min) and transient (declining to the basal level within 1 h), while the ${\text{O}}_{2}^{•-}$ generation is initially detected at 2 h and persistent (increasing for another 2 h), and the latter, but not the former, is correlated with apoptosis. In support of the role of ${\text{O}}_{2}^{•-}$, Mn(III)tetrakis(4-benzoic acid)porphyrin chloride (MnTBaP), a SOD mimetic that can scavenge ${\text{O}}_{2}^{•-}$ and ONOO^−^, but not NO, abolishes TRAIL-induced apoptosis and DHE responses (20). Moreover, analyses using MitoSOX, which is localized to mitochondria and thereby serve as a selective probe for ${\text{O}}_{2}^{•-}$ in these organelles (65), revealed that TRAIL dose-dependently induces ${\text{O}}_{2}^{•-}$ generation within the mitochondria, which is blocked by MnTBaP. ONOO^−^ can also oxidize DCFH, and MnTBaP was reported to selectively scavenge ONOO^−^ over ${\text{O}}_{2}^{•-}$ (66). However, ONOO^−^ seems to play a minor role in TRAIL-induced apoptosis, because: (i) TRAIL induces no substantial NO production for up to at least 4 h; and (ii) synthesized ONOO^−^ causes minimal cell death, although it can dose-dependently cause DCFH oxidation. The observations that MnTBaP blocks TRAIL-induced ΔΨ_m_ collapse and caspase-3/7 activation indicate that ${\text{O}}_{2}^{•-}$ is the main participator in the mitochondrial dysfunction. In addition, MnTBaP reduces TRAIL-induced XBP-1 activation (20). These observations suggest that mitochondrial ROS (mROS) mediate both mitochondrial and ER dysfunctions during TRAIL-induced apoptosis.

Studies on the effects of exogenously applied H_2_O_2_ have provided another line of evidence for the role of mROS in mediating apoptosis. Direct application of H_2_O_2_ causes apoptosis in TRAIL-resistant melanoma cells and sensitizes these cells to TRAIL (23). The potentiation of apoptosis mainly occurs through the mitochondrial and ERS pathways, as shown by ΔΨ_m_ collapse, caspase-3/7 activation, GRP-78 downregulation, and XBP-1 activation. Strikingly, after TRAIL treatment, the intracellular H_2_O_2_ level increases rapidly (within 10–30 min) but transiently (declining to the basal level at 1 h), while the intracellular ${\text{O}}_{2}^{•-}$ level increases over time for at least 4 h. MnTBaP, but not catalase, can block the ${\text{O}}_{2}^{•-}$ increase and apoptosis in parallel, indicating that ${\text{O}}_{2}^{•-}$ mainly mediates the apoptosis. In addition, a robust increase in MitoSOX signals is observed in parallel with the ${\text{O}}_{2}^{•-}$ increase and MnTBaP blocks this oxidative response, suggesting an ${\text{O}}_{2}^{•-}$ increase within the mitochondria. Moreover, H_2_O_2_ induces ΔΨ_m_ collapse, caspase-3/7 activation, and XBP-1 activation, all of which are blocked by MnTBaP treatment. These observations suggest that exogenously applied H_2_O_2_ can stimulate ${\text{O}}_{2}^{•-}$ generation within mitochondria and that the mROS mediate the mitochondrial and ER dysfunctions. In addition, H_2_O_2_ causes minimal intracellular and mROS generation in melanocytes in parallel with its minimal cytotoxic effect (23). Taken together, these findings strongly suggest a pro-apoptotic role of mROS in cancer cell apoptosis.

In normal resting cells, 0.1–2% of electrons carried by the mitochondrial electron transport chain (ETC) leak from this pathway and form ${\text{O}}_{2}^{•-}$. Impaired mitochondrial metabolism causes robust leakage of free electrons, thereby resulting in ROS generation within the organelles. Consequently, mitochondrial metabolic inhibitors serve as powerful tools for studying the role of mROS in a given biological response. Studies using Jurkat cells and A375 melanoma cells as a model have revealed that in both cell types, rotenone, an inhibitor of complex I, is the most potent at inducing apoptosis, while FCCP, a classic uncoupler of oxidative phosphorylation (OXPHOS) is less effective and complex III inhibitor antimycin A is ineffective. However, all of these compounds markedly potentiate TRAIL-induced apoptosis and mROS generation (20, 21). The three metabolic inhibitors can also potentiate TRAIL-induced activation of caspase-3/7 and XBP-1, and both effects are blocked by MnTBaP, indicating that mROS generated by the ETC mediate both mitochondrial and ER dysfunctions during TRAIL-induced apoptosis (20). These findings are similar to a previous report that CCCP, another OXPHOS uncoupler, enhances TRAIL-induced apoptosis in TRAIL-resistant human colon carcinoma cell lines (RKO, HT29, and HCT8), while CCCP alone has little effect on apoptosis or release of pro-apoptotic factors from mitochondria (67). CCCP also enhances TRAIL-induced caspase-8, Bid, caspase-9 activation, and Bax conformational change and translocation to mitochondria. CCCP treatment results in the generation of ROS as determined by DHE, and the synergistic effect of CCCP on the TRAIL-induced apoptotic pathway is abrogated by the non-specific ROS scavenger NAC. It is noteworthy that when mitochondria are uncoupled, shRNA-mediated caspase-9 depletion cannot protect the cells against cell death, suggesting modulation of the mode of the caspase cascade. It has been shown that alterations in mitochondrial function, such as OXPHOS, affect the response of tumor cells to apoptosis induced by death ligands including ${\text{O}}_{2}^{•-}$ generation (68, 69), while death ligands affect mitochondrial metabolism and function. Thus, mitochondrial function and death ligand-induced apoptosis are intimately associated with one another. In this functional relationship, loss of ΔΨ_m_, i.e., mitochondrial membrane depolarization (MMD), may play a central role, because it is not only a major cause of mROS generation but also provokes membrane integrity disruption and caspase cascade activation. There is accumulating evidence to suggest that mROS play a key role in MPT induction by affecting the mPTP conformation. First, ROS are byproducts of OXPHOS and excessive ROS generation is potentially deleterious to mitochondrial and cellular functions. Second, ANT has three cysteine residues whose oxidation is critical for mPTP open–closed transitions and Ca^2+^ release from the mitochondrial matrix, and mPTPs are believed to be particularly vulnerable to ROS (30–32). Consequently, the MPT can be triggered by excessive mROS generation and/or disruption of the mitochondrial redox homeostasis (70–73). Third, within mitochondria, cytochrome *c* is bound to the outer surface of the IMM by its association with the mitochondrial phospholipid cardiolipin, and oxidation of cardiolipin is thought to decrease this contact (74). Thus, oxidation of cardiolipin may also be required to liberate sufficient cytochrome *c* to trigger caspase activation and induce apoptosis. Consequently, oxidation of cardiolipin may serve as a biochemical hallmark of mitochondrial oxidative stress and apoptosis. As the fluorescent dye 10-*N*-acridine orange (NAO) binds to the non-oxidized, but not oxidized, form of cardiolipin independently of ΔΨ_m_, measurements of NAO fluorescence enable monitoring of the oxidation of cardiolipin in mitochondria (75). It has been shown that in parallel with the increase in MitoSOX signals, TRAIL treatment results in oxidation of cardiolipin in human leukemia and melanoma cells (20, 21). Agonistic antibodies against DR4 and DR5, which trigger the formation of multimeric complexes containing only specific TRAIL-Rs (76, 77) also induce robust cardiolipin oxidation in a dose-dependent manner, indicating that this oxidation is mediated by DR4/DR5 (21). The MPT also results in dissipation of ΔΨ_m_ and enhances mROS production via disintegration of the ETC, thereby progressively shutting down OXPHOS and impairing energetic metabolism (78). Hence, the MPT is a rate-limiting and self-amplifying process for apoptosis in which ROS and MMD play key roles. Indeed, MMD is the most common event observed concomitantly with ROS generation in cell death induced by diverse death-inducing stimuli (Tables 1 and 2).

## Crosstalk between Membrane Depolarization and ROS

**Figure 1:**
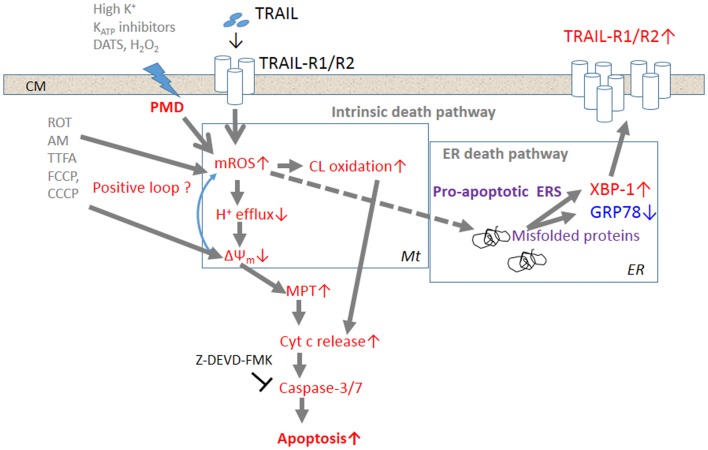
**The current model for the potentiation of TRAIL-induced apoptosis in cancer cells by depolarization**. Triggering of TRAIL-R1 (DR4)/TRAIL-R2 (DR5) induces the generation and accumulation of mROS, leading to impairment of the ETC function and cardiolipin oxidation. Impairment of the ETC complex I/III function decreases H^+^ efflux, thereby causing ΔΨ_m_ dissipation, i.e., MMD, and additional mROS generation and cardiolipin oxidation, thereby forming a positive loop. Cardiolipin oxidation and ΔΨ_m_ dissipation cooperatively promote the MPT and liberation of sufficient cytochrome *c* to trigger caspase activation and induce apoptosis. TRAIL-resistant cancer cells appear to gain considerable tolerance for oxidative stress-mediated activation of the intrinsic death pathway. Accumulation of mROS can also promote the formation of unfolded or misfolded proteins, thereby provoking ERS responses such as activation of the transcriptional factor XBP-1. XBP-1 activation leads to upregulation of the surface TRAIL-R2 expression level, thereby enhancing the death signaling. Activation of this alternative death pathway may contribute to commit TRAIL-resistant cancer cells to apoptosis.

Several lines of evidence suggest that membrane depolarization and ROS are intimately associated with one another. First, membrane depolarization controls the generation of ROS via NADPH oxidase activation. In endothelial cells (ECs), it has been shown that loss of fluid shear stress or ischemia results in the generation of ROS associated with the activation of EC NADPH oxidase (79, 80). It has been suggested that this activation of ROS generation is caused by the activation and assembly of NOX2 through phosphoinositide-3-kinase (PI3K)/serine threonine kinase Akt (81). It is noted that the activation of ROS generation is triggered by EC membrane depolarization caused by inactivation of K_ATP_ channels. It is widely accepted that cancer cells express various NOX family members (NOX 1–5, Duox 1, 2) and that these NADPH oxidases play important roles in cancer cell proliferation, death, function, and tumorigenesis (82). For instance, it has been shown that binding of SDF-1α to the chemokine receptor, cysteine (C)-X-C receptor-4, which contributes to the enhanced metastatic functions in prostate cancer cells, promotes ROS generation via NOX2 activation through the PI3K/Akt signaling pathway (83). VEGF that plays a critical role in vascular pathophysiology and induces ROS generation in B1647 cells, a human leukemia cell line, through activation of NOX2 and NOX4, and the NOX-generated ROS are required to sustain cell viability and proliferation, and prevent apoptosis (84). Second, ROS were shown to regulate the sustained membrane depolarization during tumor cell apoptosis. As mentioned above, inactivation of Na^+^–K^+^-ATPase is responsible for the sustained membrane depolarization during Fas-induced apoptosis (14, 15). This Na^+^–K^+^-ATPase inactivation was shown to be associated with cleavage of the 42-kDa β subunit and decreased levels of the 110-kDa α subunit (15). Fas ligation also triggers the internalization of plasma membrane Na^+^–K^+^-ATPase in tumor and normal cells, which is induced by intracellular glutathione depletion and H_2_O_2_ generation (85). A molecular mechanism for the H_2_O_2_-induced internalization of Na^+^–K^+^-ATPase is considered to be serine phosphorylation of the α1 subunit and regulation by mitochondrial H_2_O_2_ and the anti-apoptotic protein Bcl-2 (15, 85). Collectively, these observations suggest that ROS can control depolarization in malignant cells through the modification, degradation, and inactivation of Na^+^–K^+^-ATPase. Recently, another crosstalk between depolarization and mROS in cancer cells was suggested. Membrane-depolarizing agents by themselves increase mROS and potentiate TRAIL-induced mROS generation, indicating that depolarization controls mROS (21). It is notable that depolarization increases the surface expression of DR5, the triggering of which increases mROS. Since depolarization potentiates TRAIL-induced activation of the transcription factor XBP-1, which is engaged in the regulation of surface DR5 expression (26), it is possible to speculate that the upregulation of surface DR5 expression results in increased mROS accumulation. However, scavenging of mROS by the antioxidant MnTBaP reduces depolarization, while mROS accumulation caused by mitochondrial metabolic dysfunction potentiates the depolarization (21). Collectively, these observations suggest that depolarization and mROS mutually regulate one another. Figure 1 shows the current model for the potentiation of TRAIL-induced apoptosis in cancer cells by membrane depolarization in connection with ROS.

Several TRAIL-resistant cancer cell types, such as malignant melanoma cells, appear to gain considerable tolerance for oxidative stress-mediated activation of the intrinsic death pathway. However, accumulation of mROS can also promote the formation of unfolded or misfolded proteins, thereby provoking ERS responses such as activation of the transcriptional factor XBP-1. XBP-1 activation leads to upregulation of the surface TRAIL-R2 expression level, thereby enhancing the death signaling. When used together with TRAIL, membrane-depolarizing agents can potentiate mROS accumulation sufficiently to activate not only the intrinsic death pathway, but also the ERS-mediated death pathway, thereby committing drug-resistant cancer cells to apoptosis. Although the precise biochemical and biological consequences between membrane depolarization and mROS generation remain to be elucidated, these two events occur in parallel, indicating that they are intimately associated. Since TRAIL signaling alone can induce sustained depolarization, it is possible that the ROS increase causes this response by inactivating plasma membrane Na^+^–K^+^-ATPase and/or K_ATP_ channels.

## Abnormal Increases in ROS in Cancer Cells – A Target for Selective Killing

**Figure 2:**
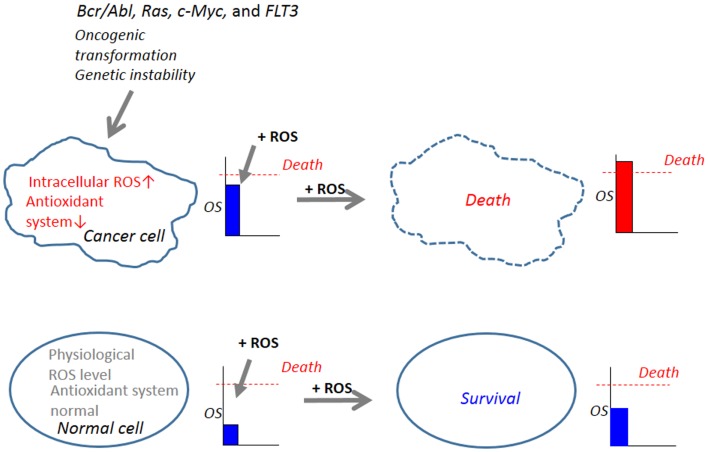
**Abnormal increases in ROS in cancer cells serves as a target for tumor-selective killing**. Cellular oxidative stress (OS) level is regulated by the balance between the machinery for ROS generation (prooxidant system) and the machinery for ROS scavenging (antioxidant system). In normal cells, the antioxidant system is normal and low physiological levels of ROS, which can function as second messengers in intracellular signaling and are required for normal cell function, are generated. Owing to their active metabolism and genetic instability under the control of oncogenic transformation such as *Bcr/Abl, Ras, c-Myc*, and *FLT3*, which causes increased ROS generation and decreased antioxidant systems, cancer cells harbor an excess OS over normal cells. When equivalent levels of OS are added by the administration of exogenous ROS-inducing agents (+ROS), OS levels in cancer cells can readily over the threshold of cell death, while OS levels in normal cells do not. Hence, cancer cells are expected to be more vulnerable than normal cells to cell damage induced by ROS-generating agents and this vulnerability can be exploited to selectively kill these cells.

The steady-state levels of endogenous ROS including ${\text{O}}_{2}^{•-}$ and H_2_O_2_ are elevated in cancer cells (86, 87). The increase in ROS is thought to contribute to maintenance of the cancer cell phenotype through their effects on cell growth, proliferation, and genetic instability (88, 89). Increased ROS generation is common in cancer cells with active metabolism and genetic instability under the impact of various oncogenes such as *Bcr/Abl, Ras, c-Myc*, and *FLT3*, FMS-like tyrosine kinase (90–95). The imbalance between the prooxidant system and the antioxidant system is a consequence of increased ROS generation and/or decreased antioxidants in cancer cells. Such abnormal increases in ROS makes cancer cells more vulnerable than normal cells to cell damage induced by exogenous ROS-inducing agents and can be exploited to selectively kill cancer cells (96–98) (Figure 2). In this respect, it is noted that diverse natural products and synthetic chemicals that exhibit selective killing effects are capable of inducing intracellular ROS generation (Tables 1 and 2). For instance, β-phenylethyl isothiocyanate (PEITC) is a natural product found in cruciferous vegetables with chemopreventive activity, and has been shown to increase ROS generation and induce apoptosis (99, 100). Moreover, recent work has shown the selective killing of oncogenically transformed cells by PEITC (101–103), supporting the emerging view that an intrinsic excess oxidative stress under the control of oncogenic transformation can be exploited as a cancer-selective target.

## Conclusion

Recent work has revealed that membrane depolarization is an early and prerequisite event during death ligand-induced apoptosis in malignant cells. Moreover, it has been shown that persistent membrane depolarization can facilitate tumor-selective killing and TRAIL-sensitizing effects by promoting mitochondrial and ER dysfunctions. It is noted that membrane depolarization and ROS regulate one another although the precise mechanisms underlying the mutual regulation remain to be elucidated. The emerging concept is that owing to their active metabolism and genetic instability under the control of oncogenic transformation, cancer cells harbor an excess oxidative stress over normal cells and that this intrinsic oxidative stress can be exploited to selectively kill malignant cells. Thus, the crosstalk is one possible rationale why diverse membrane-depolarizing agents can exhibit tumor-selective killing and TRAIL-sensitizing effects in cancer cells. Since depolarization is intimately linked to not only mitochondrial integrity disruption but also ER dysfunction, and impacts cancer cells selectively, further studies on its role in cancer cell death may afford a novel approach for tumor-selective killing.

## Conflict of Interest Statement

The authors declare that the research was conducted in the absence of any commercial or financial relationships that could be construed as a potential conflict of interest.

## Abbreviations

AIF, apoptosis-inducing factor; ANT, adenine nucleotide translocase; Apaf-1, apoptotic peptidase activating factor 1; ATF6, activating transcription factor 6; DADS, diallyl disulfide; DAS, diallyl sulfide; DATS, diallyl trisulfide DCFH-DA, 2′,7′-dichlorofluorescein diacetate; DcR, decoy receptor; DHE, dihydroethidine; DR, death receptor; ΔΨ_m_, mitochondrial membrane potential; EC, endothelial cell; eIF2, eukaryotic initiation factor-2; ER, endoplasmic reticulum; ERK, extracellular signal-regulated protein kinase; ERS, endoplasmic reticulum stress; ETC, electron transport chain; FasL, Fas ligand; GRP78, glucose-related protein 78; IMM, inner mitochondrial membrane; IP_3_, inositol-1,4,5-triphosphate; IRE1, inositol requiring enzyme 1; JNK, Jun-N-terminal kinase; K_ATP_, ATP-sensitive K^+^; K_Ca_, calcium-dependent K^+^; K_v_, voltage-dependent K^+^; mAbs, monoclonal antibodies; MHP, mitochondrial hyperpolarization, MMD, mitochondrial membrane depolarization; MnTBaP, Mn(III)tetrakis(4-benzoic acid)porphyrin chloride; MPT, mitochondrial permeability transition; mPTP, mitochondrial permeability transition pore; mROS, mitochondrial reactive oxygen species; NAC, *N*-acetyl-l-cysteine; NAO, 10-*N*-acrydine orange; NSCLC, non-small cell lung cancer; OMM, outer mitochondrial membrane; OXPHOS, oxidative phosphorylation; PARAs, pro-apoptotic receptor agonists; PEITC, β-phenyethyl isothiocyanate; PI3K, phosphoinositide-3-kinase; PMD, plasma membrane depolarization; rh, recombinant human; ROS, reactive oxygen species; siRNA, small interfering RNA; SMAC, second mitochondrial activator of caspases; SOD, superoxide dismutase; SUR, sulfonylurea receptor; TEA, tetraethylammonium; TNF, tumor necrosis factor; TRAIL, TNF-related apoptosis-inducing ligand; TRAIL-R, TNF-related apoptosis-inducing ligand receptor; TRPV1, transient receptor potential vanilloid 1; UPR, unfolded protein response; VDAC, voltage-dependent anion channel; VEGF, vascular endothelial growth factor; XBP-1, X-box-binding protein-1; XIAP, X-linked inhibitor of apoptosis protein.
